# Money demand under free banking: Switzerland 1851–1906

**DOI:** 10.1186/s41937-017-0013-8

**Published:** 2018-03-20

**Authors:** Stefan Gerlach, Peter Kugler

**Affiliations:** 1 BSI Bank, Schützengasse 31, 8021 Zürich, Switzerland; 2 CEPR, London, UK; 3 WWZ, University of Basel, Peter Merian-Weg 6, CH-4002 Basel, Switzerland

**Keywords:** Free banking, Money demand, Monetary dynamics, Switzerland, E41, E42, N13

## Abstract

This paper studies money demand in Switzerland under free banking before the establishment of the Swiss National Bank. We find that, in addition to income, the banks’ balance-sheet-to-GDP ratio and the number of banks were important determinants of long-run money demand. The former variable also played an important role in the monetary adjustment process. We also detect a strong positive long-run impact of real income on the bank’s balance-sheet-total-to-GDP ratio and a strong long-run influence of real income and the interest rate spread on the number of banks.

## Background

Before the foundation of Swiss National Bank in 1907, banknotes were issued by private banks in Switzerland. Thus, all components of M1, except coins, were issues by private banks under a regime of free banking. Under such circumstances, an increase in the money supply may occur at the extensive margin through an increase in the number of banks or at the intensive margin through an increase in the money supply by existing banks. Moreover, the years since the creation of the Swiss franc as a national currency in 1850 witnessed a strong change in the composition of the M1. In 1851, approximately 87% of M1 were coins and the rest was shared by banknotes and sight deposits. In 1906, the share of sight deposits increased to 74% and that of coins shrank to 7% (Baltensperger and Kugler 2015a, 2015b, Chapter III.1). This development is mirrored on the one hand by an increase in the number of banks from 520 in 1851 to 610 in 1880, a level which remained largely constant thereafter. On the other hand and more importantly, the aggregate bank balance sheet total was six times larger in 1906 than in 1851. Switzerland thus underwent a substantial change in financial structure and a rapid development of banking during this period which needs to be taken into account in analyzing money demand and monetary dynamics.

The rapid financial development of the US compared to the financially highly developed UK economy in the nineteenth century was discussed by Friedman and Schwartz (1982). They found that from 1867 to 1903 increased financial sophistication in the USA lead to a long-run increase of M1 beyond the growth of nominal income. Based on their analysis, they calculated an adjusted US money stock attempting to put into account financial developments. In this paper, we follow a different approach by including the developments of the banking sector directly into the analysis of money demand and monetary dynamics in Switzerland from 1851 to 1906.

There are two reasons for why doing so is interesting. First, an increase in the number of banks and in their activities has a direct effect on money demand as banknotes and sight deposits become more attractive by increased bank coverage of a country. One would thus expect that the growing activity of banks increased the demand for money. Whether or not this is so is an empirical question that, as far as we know, has not yet been analyzed econometrically.

Second, Baltensperger and Kugler (2017, Chapter 9) estimate money demand functions for Switzerland before WW I and report a point estimate of 1.62 for the income elasticity. After the WW I, however, the income elasticity is not significantly different from 1 and exhibits an impressive stability over time. Since the growth of the banking system occurred in a period of strong income growth, one obvious possibility is that high-income elasticity estimated by Baltensperger and Kugler results from the fact that they do not incorporate the growth of the banking system in their analysis. As a consequence, its importance for money demand is instead attributed to income growth.

The paper is organized as follows: the “Free banking in Switzerland 1850–1905/1907” section gives a brief account of the development of free banking between the creation of the Swiss franc in 1850 and the establishment of the Swiss National Bank in 1905–1907. This period started with nearly unregulated competition in banknote issue, but the harmonization of privately issued banknotes required by federal banking law of 1881 undermined this system and finally led to the creation of SNB.

The “A simple model of money demand under free banking” section then considers a long-run model of money demand in which we take into account explicitly the banking activity in a model with four cointegrating relations. Money demand depends on the ratio of the bank balance-sheet-total-to-GDP and the number of banks as additional variables. The ratio of balance-sheet-total-to-GDP depends on real income. The same applies to the number of banks which depend in addition on the spread between the interest rates for mortgage loans and savings deposits which is also governed by a long-run relationship. Our empirical results are reported in the “Empirical results” section. The main finding is that banking activity is an important variable in long-run money demand as well as in the monetary adjustment process under free banking. Finally, the “Conclusions” section concludes.

## Free banking in Switzerland 1850–1905/1907

The components of M1 (coins, banknotes, and sight deposits) were issued under different regimes before the foundation of Swiss National Bank.^1^ In 1850, Switzerland created the Swiss franc as the national currency of the new federal state founded in 1848 with common coins. Switzerland stuck to a competitive solution for banknote issue until 1907, when the Swiss National Bank started operating. The authorization of banks and their regulation remained with the cantons and differed widely. Nevertheless, a crucial break took place in 1881, when the previously unregulated regime of competition in banknote issue was followed by the heavily regulated regime of the new Federal Banknote Act. As far as the creation of money in the form of bank deposits is concerned, Switzerland, like virtually all other countries, has stayed with a competitive system until today.

The monetary reform of 1850 brought an important change. After its introduction, the Swiss franc established itself without problems and quickly as the new federal state’s national currency. Legally, Swiss banks were still allowed to issue notes denominated in foreign currencies. A few banks did make use of this possibility for some time, in addition to issuing notes in Swiss francs. However, with the Swiss franc now firmly established as the dominant national currency, the demand for foreign currency notes more or less vanished; in consequence, the practice was soon terminated.

The period from 1850 to 1881 thus can be characterized as a period of free, unregulated note issue competition, but now with a common, dominant currency, the Swiss franc. The authority to license and regulate banks still belonged to the cantons as no federal restrictions existed before 1881. The cantons were liberal in permitting new banks and in regulating their business. As a consequence, a large number of note issuing banks competing with each other entered the market, some private and some public, the latter in the form of cantonal banks established by the cantons themselves. In 1880, no less than 36 note issuing banks existed.^2^ The cantonal banks were not granted a monopoly or other privileges by their cantons, so the note issue business remained highly competitive.

This system was successful in the sense that it provided banknotes with stable purchasing power while (almost) avoiding financial turbulence and bank failures (Weber, 1988 and 1992).^3^ The issue of banknotes involved considerable costs: production (printing) costs, personnel costs of bank counter service and, particularly important, the costs of the metal reserves necessary for reasons of confidence and trust, and the costs of banknote clearing. Note clearing was a central element of concern: the demand for banknotes was dependent on their acceptance at full value which led the formation of clearing networks including entire groups of banks. For all these reasons, banks’ note issue business remained small and of limited importance throughout most of this period. Complaints about the complicated nature and the inefficiency of the payments’ system remained frequent. Several authors, e.g., Jöhr (1915) or Ritzmann (1973), stressed this state of dissatisfaction and the inefficiencies causing it and thus came, in contrast to Weber, to a negative assessment of note issue competition in this period. The Federal Banknote Act of 1881, introducing common standards of quality in banknote issue, was the result of these perceived inefficiencies.

The period from 1881 to 1905 can be characterized as a period of strictly limited banking freedom: a system with now legally prescribed currency denomination for banknotes and a heavily regulated and harmonized note issue business, but still without a centralized government monopoly in note issue. Banks were severely constrained with regard to their liquidity reserves, their equity capital, their banknote redemption, and their issuing policies. The regulatory standardization and mutual acceptance of notes issued by different banks improved the efficiency of the money and payments system. These conditions, however, undermined competition as it introduced an externality creating incentive to an over-issue of banknotes leading to a tendency towards monetary and currency weakness^4^—a state of affairs which would ultimately lead to full nationalization of banknote issue and the foundation of the Swiss National Bank. In 1891, a revision of the constitution was accepted by the voters which introduced the exclusive right of the confederation to issue banknotes. Conflicts about the legal form of the central bank and the location of its headquarter implied that the Federal Act on the Swiss National Bank came into effect only 14 years later in October 1905. The SNB started its operation in June 1907.

**Fig. 1:**
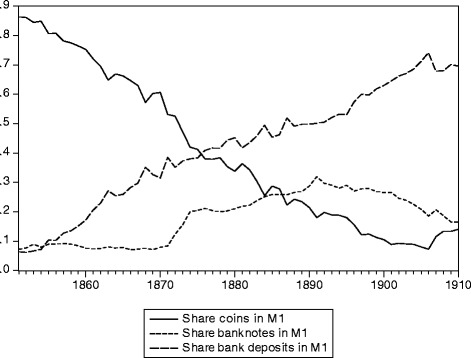
The composition of M1 in Switzerland 1851–1906. Data source: Swiss economic and social history database: M1 1851–1907 (Table Q3), http://www.fsw.uzh.ch/hstat/hsso/overview.php

Figure 1 depicts the development of the shares of coins, banknotes, and sight deposits in M1 over the period 1851–1906. This figure shows that Switzerland was a “coin economy” at the time of the introduction of the Swiss franc: approximately 87% of M1 were coins in 1851 and the remaining 13% were shared equally by banknotes and sight deposits. In the following years, we observe a trend decline (increase) in the roles of coin (sight deposits) resulting in shares of 7 and 74% in 1906, respectively. By contrast, the development of the banknote share is less monotonic: it experienced a strong increase in the 1870s in the aftermath of the liquidity crisis triggered by the inconvertibility of the French franc during the Prussian-French war. It increased again after the switch to federal regulation of banknote issue (in 1881) and reached a peak of 30% in 1891. This illustrates the incentives to over-issue for banks as mentioned above. The weakness of the Swiss franc at the foreign exchange market and the associated loss of monetary metal and increasing costs of banknote issue led to a decline of the banknote share to 19% in 1906.

## A simple model of money demand under free banking

Let *m*_*t*_, *y*_*t*_, *p*_*t*_, *bb*_*t*_, *nb*_*t*_, *rs*_*t*_, and *rm*_*t*_ denote the logarithm of M1, real income, the price level, and the balance sheet total of banks and the number of banks as well as the level of the interest rates on savings deposits and mortgage lending, respectively. The two interest rates were selected as they are the only relevant rates which are consistently available for our sample period. We start with a modified money demand function that includes banking activity measured by the balance sheet total relative to GDP and the number of banks as an additional demand factor. Omitting the intercept term, we have

1 $$ {m}_t={p}_t+{y}_t+{b}_1{rs}_t+{b}_2\exp \left({bb}_t-{y}_t-{p}_t\right)+{b}_3{nb}_t+{u}_{1t}, $$

where *u*_1*t*_ is a stationary and auto-correlated deviation from long-run money demand and all parameters are expected to be positive except the interest rate semi-elasticity, *b*_1_. Note that we restricted the (real) income elasticity to 1 which appears to be appropriate for Switzerland since the early twentieth century (Baltensperger and Kugler 2017, Chapter 9).

The use of the ratio of balance-sheet-total-to-GDP is advisable for theoretical and empirical reasons even if it makes the model nonlinear. Financial development measured by this indicator exhibits a strong positive long-run relationship with real income:

2 $$ \exp \left({bb}_t-{y}_t-{p}_t\right)={b}_4{y}_t+{u}_{2t}, $$

where *u*_3*t*_ is a stationary and auto-correlated deviation from long-run equilibrium.

The number of banks is considered an endogenous variable, too, and depends positively on real income as an indicator of economic activity and the spread between the credit and the savings rates as an indicator for the profitability of banking. Omitting the constant, we have

3 $$ {nb}_t={b}_5{y}_t+{b}_6\left({rm}_t-{rs}_t\right)+{u}_{3t}, $$

where *u*_2*t*_ is a stationary and auto-correlated deviation from long-run equilibrium and all parameters are expected to be positive.

Moreover, we add long-run relationship between the two interest rates:

4 $$ {rm}_t={b}_7\ {rs}_t+{u}_{4t}. $$

If *b*_7_ differs from 1, the interest rate spread is nonstationary and two non-cointegrated I(1) variables enter Eq. (3).

This simple model immediately shows the effect of estimating a standard money demand function omitting the banking activity: the positive correlation of the omitted variable with real income leads to a positive bias of the estimate of the income elasticity of money demand. Below, we explore if the high-income elasticity estimated of Baltensperger and Kugler (2017, Chapter 9) arises for this reason.

## Empirical results

### Unit root and cointegration properties

**Fig. 2:**
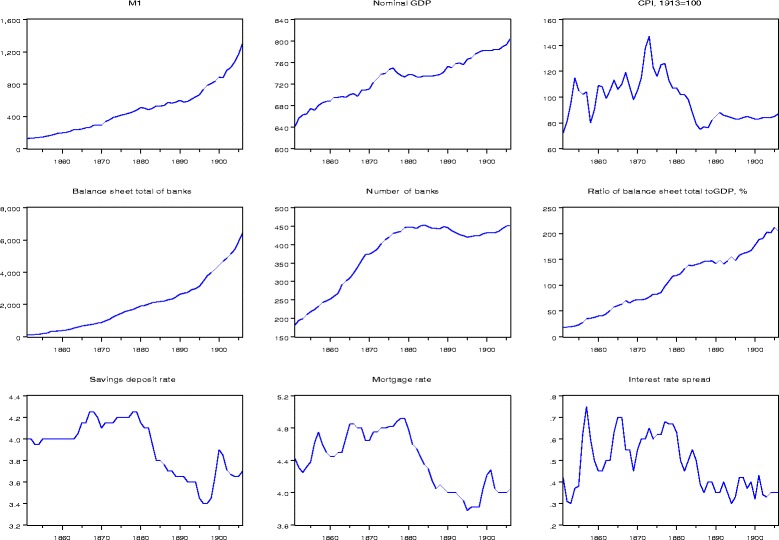
Data series Swiss money demand estimation, 1851–1906. Nominal values in million SFr, interest rates in %. Data source: Swiss economic and social history database: M1 (Table Q3), GDP (Table Q16a, b), number of banks (Q8), and CPI (H17); http://www.fsw.uzh.ch/hstat/hsso/overview.php. SNB historical series: saving deposit and mortgage interest rates, https://www.snb.ch/de/iabout/stat/statrep/statpubdis/id/statpub_histz_arch#t3

In this section, we show the annual data for our analysis and provide the unit root analysis for the Swiss money stock M1, nominal income (GDP), price level (CPI), bank balance sheet total, number of banks, the savings interest rate, and the interest rate on mortgages, the only bank credit interest rate which is available for the nineteenth century. Note that all variables except interest rates are in logs and are multiplied by 100 (so that their first differences can be thought of as percent changes) for the econometric analysis. Moreover, because of lack of real GDP data for the entire period, we have to consider nominal GDP and use the consumer price index in order to obtain real GDP for model estimation. The (unlogged) series as well as the balance-sheet-total-to-GDP ratio and the interest rate spread are plotted in Fig. 2.

All variables except interest rates have an obvious trend. Therefore, we run unit root and stationarity tests including a deterministic trend for log M1, log GDP, log CPI, log balance sheet total, and the number of banks. Table 1 contains the results of the Phillips-Perron unit root test and Kwiatkowski-Phillips-Schmidt-Shin stationarity test.

**Table 1:** Unit root and stationarity tests, 1851–1906

Series	PP	PP with trend	KPSS	KPSS with trend
Log M1		− 1.631		0.183**
Log CP1		− 3.124*		0.148**
Log GDP		− 2.960		0.118
Log bank balance sheet total		− 3.254		0.241***
Log number of banks		− 2.991		0.241*
Ratio of balance-sheet-total-to-GDP		− 2.645		0.059
Savings rate	− 1.109		0.551**	
Mortgage rate	− 1.024		0.554**	
Interest rate spread	− 1.907		0.476**	

Notes: Lag length for the nonparametric autocorrelation correction selected automatically according to Newey-West and Bartlett kernel

*Significant at the 10% level, **significant at the 5% level, ***significant at the 1% level

Overall, Table 1 indicates that the series are I(1) even if we get mixed results for two series. In five cases (M1, balance sheet total, number of banks, and two interest rates), the unit root hypothesis cannot be rejected at the 5% level and the stationarity hypothesis is always rejected at the 5% level. For nominal GDP and the ratio of balance-sheet-total-to-GDP, neither the unit root nor the trend-stationarity hypothesis can be rejected at standard significance levels and the data are inconclusive. For the CPI, we reject the unit root hypothesis on the 10% level and the stationarity hypothesis at the 5% level.

We estimated the cointegration coefficients using fully modified ordinary least squares. This approach is an obvious choice given the nonlinearity introduced by the unlogged ratio of balance-sheet-total-to-GDP in Eq. (1) which prevents the application of the Johansen approach. Moreover, we avoid a potential bias of the cointegration estimates obtained by the Johansen’s EC system approach caused by a possible structural break in the adjustment process.

The cointegration analysis clearly points to the existence of such a cointegrating long-run money demand function as a Hansen test does not reject the null hypothesis of cointegration at any reasonable significance level. In addition, the null hypothesis of no cointegration is rejected at the 5% significance level by the Phillips-Ouliaris test. The estimated interest elasticity had a “wrong” positive sign and was completely statistically insignificant. Therefore, we set it to zero. The balance-sheet-total-to-GDP ratio appears as a strong indicator representing the effect of banking development during our sample period. The number of banks is indicated to be important for money demand, albeit with a clearly lower statistical significance. The ratio of bank balance sheet total is indicated to be clearly cointegrated with real income as the Hansen (Phillips-Ouliaris) test does not reject (rejects). The cointegration tests for the number of banks provides the same pattern of results: the hypothesis of no cointegration is rejected by the Phillips-Ouliaris test at the 10% level and the null of cointegration is not rejected by the Hansen test. The coefficient of the interest spread and real income are positive and statistically highly significant. Finally, the cointegration analysis for the two interest rates delivers clear-cut results: the null of no cointegration can be clearly rejected whereas the null of cointegration is not rejected. The change in the mortgage rate is estimated to be roughly 1.44 times that of the savings rate in the long run (Table 2).

**Table 2:** Cointegration test and estimates, M1, real income, price level, interest rates, and bank balance sheet total, Switzerland, 1851–1906

*m*_*t*_ = *p*_*t*_ + *y*_*t*_ + *b*_1_*rs*_*t*_ + *b*_2_ exp(*bb*_*t*_ − *y*_*t*_ − *p*_*t*_) + *b*_3_*nb*_*t*_ + *u*_1*t*_ exp(*bb*_*t*_ − *y*_*t*_ − *p*_*t*_) = *b*_4_*y*_*t*_ + *u*_2*t*_, *bb*_*t*_ − *p*_*t*_ = *b*_5_*y*_*t*_ + *b*_6_(*rm*_*t*_ − *rs*_*t*_) + *u*_3*t*_, *rm*_*t*_ = *b*_7_ *rs*_*t*_ + *u*_4*t*_.										
Equation	*b*_2_	*b*_3_	*b*_4_	*b*_5_	*b*_6_	*b*_7_	R^2^	DW	Phillips-Ouliaris Test	Hansen Test
*m-p-y*	0.352*** (0.0317)	0.220** (0.067)					0.970	0.634	− 4.547**	0.201
exp(*bb-y-p*)			1.221*** (0.120)				0.955	0.794	− 4.020**	0.234
*bn*				0.629*** (0.0796)	130.92*** (28.883)		0.700	0.553	− 3.327*	0.219
*rm*						1.440*** (0.0620)	0.938	0.843	− 3.874**	0.244

Standard errors in parenthesis

*Significant at the 10% level, **significant at the 5% level, ***significant at the 1% level

### EC model estimates

In this section, we present the estimates of the standard EC model for our six variables which can be written as^5^

5 $$ {\displaystyle \begin{array}{l}\Delta {x}_{it}={\gamma}_{i1}{u}_{1t-1}+{\gamma}_{i2}{u}_{2t-1}+{\gamma}_{i3}{u}_{3t-1}+{\gamma}_{i4}{u}_{4t-1}+{\sum}_{j=1}^7{c}_{ij}\Delta {x}_{jt-1}+{\varepsilon}_{it},\kern1.75em i=1,2,\dots 7\\ {}\Delta x{\hbox{'}}_t=\left(\Delta {m}_t,\Delta {p}_t,\Delta {y}_t,\Delta {rs}_t,\Delta {bb}_t,\Delta {nb}_t,\Delta {rm}_t\right).\end{array}} $$

Table 3 shows estimates, which have mostly the expected sign, in particular when they are statistically significant. We see that an excess money stock (deviation from long-run money demand) is corrected gradually by itself (ca 26% annually) and an increase in the price level (ca 47%) whereas the other variables are not directly affected by excess money.

**Table 3:** EC model estimates M1, real income, price level, interest rates, and number of banks, Switzerland, 1851–1906

*∆x*′_*t*_ = (*∆m*_*t*_, *∆p*_*t*_, *∆y*_*t*_, *∆rs*_*t*_, *∆bb*_*t*_, *∆nb*_*t*_, *∆rm*_*t*_) ${\Delta x}_{it}={\gamma}_{i1}{u}_{1t-1}+{\gamma}_{i2}{u}_{2t-1}+{\gamma}_{i3}{u}_{3t-1}+{\gamma}_{i4}{u}_{4t-1}+\sum \limits_{j=1}^7{c}_{ij}{\Delta x}_{jt-1}+{\varepsilon}_{it},\kern1.75em i=1,2,\dots 7$						
Equation	*γ*_i1_	*γ*_i2_	*γ*_i3_	*γ*_i4_	Adj *R*^2^	se
*∆m*_*t*_	− 0.259** (0.108)	0.0551 (0.0327)	− 0.105** (0.046)	− 4.017 (6.659)	0.0700	3.542
*∆p*_*t*_	0.471** (0.193)	− 0.336*** (0.0795)	− 0.129 (0.0961)	− 30.186** (15.016)	0.348	6.881
*∆y*_*t*_	− 0.226 (0.243)	0.355*** (0.103)	0.0931 (0.114)	23.565 (18.960)	0.271	7.983
*∆rs*_*t*_	0.000326 (0.00190)	− 0.001295 (0.000804)	0.00011 (0.000905)	0.390** (0.132)	0.291	0.0601
*∆bb*_*t*_	0.150 (0.165)	− 0.0914* (0.0480)	− 0.0298 (0.0585)	− 22.846** (9.587)	0.486	3.650
*∆nb*_*t*_	− 0.00174 (0.0525)	0.00682 (0.0278)	− 0.0251 (0.0248)	− 2.943 (4.188)	0.401	1.586
*∆rm*_*t*_	− 0.0000413 (0.00277)	− 0.00325*** (0.00119)	0.000299* (0.00155	− 0.376* (0.231)	0.210	0.0871

Note: Standard errors are given in parentheses

*, **,*** indicates significance at the 10, 5 and 1% level, respectively

A positive deviation of the balance-sheet-total-to-GDP ratio corrects itself gradually (ca 9% per annum) and is corrected by an increase in real income. Interestingly, “too high level of bank activity” has a negative effect on the price level (leading only to a tiny increase in nominal GDP) and has a strong effect negative impact on the mortgage interest rate.

A “too large number of banks” has a negative impact on the money stock and a positive one on the mortgage rate, which is however only weakly statistically significant.

Finally, a too high mortgage rate corrects itself quickly (ca 38% per annum) and is in addition corrected by an increase in the savings rate of approximately the same size. Moreover, it has a strong negative effect bank balance sheet total and the price level.

In sum, our results indicate an important role of banking activity measured by the balance-sheet-total-to-GDP ratio as determinant of the long-run money demand as well as in the monetary adjustment process under free banking. First, we found a highly statistically and economically significant positive effect of this banking activity variable and of the number of banks on money demand in addition to those of nominal income. Second, four of our seven variables react statistically highly significantly to a disequilibrium with respect to the balance-sheet-total-to-GDP ratio. Moreover, the adjusted *R*^2^ is clearly the highest for the error-correction equation for the balance sheet total (0.49) indicating a strong and highly significant reaction of banking activity to changes in the other variables.

Finally, we checked the stability over time of our model. The obvious reason for such an analysis is the fundamental institutional and regulatory change for the banknote issue business by the Federal banking act of 1881 which could have changed monetary dynamics.

Table 4 reports the results of the Chow and the Quandt-Andrews stability tests for the six EC equations. For most EC equations, the Chow test with break date 1881 points to no structural break: only for the two interest rates, we can reject the null hypothesis of stability at the 5% level. This finding is confirmed by the Andrews-Quandt test with an unknown break date: for all equations except that for the balance sheet total, no significant break can be found. In the latter case, the maximum *F*-statistics indicates a break in 1870, more than 10 years before the federal law on banknote issue and the estimation of the EC equation for the two sub-samples, which suffers evidently from low degrees of freedom and shows no striking difference between the coefficient estimates. Thus, we have no clear pattern of instability of the EC equations and we can rely on the results shown in Tables 2 and 3.

**Table 4:** Stability tests for the single EC equations 1851–1906

Equation	Chow *F*-statistics 1881	Quandt-Andrews maximum *F*-statistics	Break year Q-A test
*∆m*_*t*_	0.746	1.307	1870
*∆p*_*t*_	1.504	1.626	1878
*∆y*_*t*_	1.014	1.138	1879
*∆rs*_*t*_	1.846*	2.171*	1883
*∆bb*_*t*_	1.239	4.806***	1870
*∆nb*_*t*_	0.993	2.101	1870
*∆rm*_*t*_	1.379	1.690	1882

*Significant at the 10% level, ***significant at the 1% level

## Conclusions

In this paper, we have studied the demand for money and monetary adjustment in Switzerland before the establishment of the Swiss National Bank, which started operations in 1907. We draw two main conclusions.

First, the money stock M1, banking activity measured by the balance-sheet-total-to-GDP ratio, the number of banks, the mortgage rate, real income, the price level and the savings rate are connected by four cointegrating relations. Besides nominal income (with unit elasticity), banking activity and the number of banks enter the equation for long-run money demand with an estimated semi-elasticity of 0.35 and an elasticity of 0.22, respectively. Moreover, banking activity is highly significantly cointegrated with real GDP, and the number of banks is governed by a long-run relationship showing a positive dependence on real GDP and the interest spread. The change in the mortgage rate is indicated to be roughly 1.44 times that of the savings rate in the long run.

Second, four of our seven variables react statistically highly significantly to a disequilibrium with respect to the balance-sheet-total-to-GDP ratio. Moreover, the adjusted *R*^2^ is clearly the highest for the EC equation for the balance-sheet-total-to-GDP ratio (0.49) indicating a strong and highly significant reaction of banking activity to changes in the other variables. The number of banks plays a lesser role in the adjustment process. Moreover, its structural break tests indicate that monetary dynamics was not strongly affected by the Federal Banking Law of 1881 which changed the institutional framework for banknote issue considerably.

Overall, our empirical analysis of the Swiss experience with free banking in the nineteenth century points to an important role of the banking activity as determinant of the long-run money demand as well as in the monetary adjustment process under in an economy with rapid monetary and financial development.

## Footnotes

1 This section summarizes the analysis of Baltensperger and Kugler (2017, Chapter 8).

2 Jöhr (1915) presents a comprehensive list of all Swiss note issuing banks between 1825 and 1906; see Table III.3.

3 Switzerland was not totally exempted from banking problems in this period, though. However, these problems had little to do with banks’ note issue business. In 1870, the Banque Cantonale du Valais failed as a result of the financing of public (cantonal) deficits and bad investments (causing a political scandal involving the resignation of several cantonal government members). The Banque Générale Suisse, founded in 1853 in Geneva after the model of the French Crédit Mobilier, had to suspend payments in 1859 and was liquidated in 1869. The Eidgenössische Bank, founded in 1864 in Berne, had to be restructured by the end of the 1870s.

4 This weakness of the Swiss Franc, in particular against the French Franc, with relatively high and positive deviations from metallic parity is documented by Baltensperger and Kugler (2016).

5 The Akaike criterion as well as a sequential likelihood ratio test indicates that a lag length of 1 is optimal for the system, whereas the Schwarz and Hannan-Quinn criteria indicate a value of 1. As some of the first difference terms are highly significant, we adopted the former lag length.
